# Evaluation of a Direct Reverse Transcription Loop-Mediated Isothermal Amplification Method without RNA Extraction for the Detection of Human Enterovirus 71 Subgenotype C4 in Nasopharyngeal Swab Specimens

**DOI:** 10.1371/journal.pone.0052486

**Published:** 2012-12-18

**Authors:** Kai Nie, Shun-xiang Qi, Yong Zhang, Le Luo, Yun Xie, Meng-jie Yang, Yi Zhang, Jin Li, Hongwei Shen, Qi Li, Xue-jun Ma

**Affiliations:** 1 State Key Laboratory for Molecular Virology and Genetic Engineering, National Institute for Viral Disease Control and Prevention, Chinese Center for Disease Control and Prevention, Beijing, People's Republic of China; 2 Institute for Viral Disease Control and Prevention, Center for Disease Control and Prevention of Hebei, Shijiazhuang, Hebei, People's Republic of China; Lady Davis Institute for Medical Research, Canada

## Abstract

Human enterovirus 71 (EV71) is the major causative agent of hand, foot, and mouth disease (HFMD) worldwide and has been associated with neurological complications which resulted in fatalities during recent outbreak in Asia pacific region. A direct reverse transcription loop-mediated isothermal amplification (direct RT-LAMP) assay using heat-treated samples without RNA extraction was developed and evaluated for the detection of EV71 subgenotype C4 in nasopharyngeal swab specimens. The analytical sensitivity and specificity of the direct RT-LAMP assay were examined. The detection limit of the direct RT-LAMP assays was 1.6 of a 50% tissue culture infective dose (TCID_50_) per reaction and no cross-reaction was observed with control viruses including Cosackievirus A (CVA) viruses (CVA2,4,5,7,9,10,14,16, and 24), Coxsackievirus B (CVB) viruses (CVB1,2,3,4, and 5) or ECHO viruses (ECHO3,6,11, and 19). The direct RT-LAMP assay was evaluated and compared to both RT-LAMP and quantitative real-time PCR (qRT-PCR) in detecting EV71 infection with 145 nasopharyngeal swab specimens. The clinical performance demonstrated the sensitivity and specificity of direct RT-LAMP was reported to be 90.3% and 100% respectively, compared to RT-LAMP, and 86.83% and 100% respectively, compared to qRT-PCR. These data demonstrated that the direct RT-LAMP assay can potentially be developed for the point of care screening of EV71 infection in China.

## Introduction

Human enteroviruses (HEV) comprise more than 100 serotypes in four species (HEV-A to HEV-D) in the genus Enterovirus, family Picornaviridae. Hand, foot, and mouth disease (HFMD) is a common febrile illness in young children and is characterized by lesions on the skin and oral mucosa. HFMD cases caused by EV71 infections have been found to be associated with severe neurological complications [1].

Routine methods for EV71 detection are virus isolation, neutralization, and quantitative real time PCR (qRT-PCR) [1], [2], [3]. A few commercial qRT-PCR diagnostic kits for EV71 are available and approved by the State Food and Drug Administration of China and have been widely used in Center for disease control and prevention (CDC) of provincial and municipal regions in China for HFMD pathogens surveillance. However, these methods either are with low specificity and sensitivity (virus isolation and neutralization) or need large expense for equipments and a relative long reaction time (qRT-PCR).

Loop-mediated isothermal amplification (LAMP) is a nucleic acid amplification method first described in 2000 [4], it has emerged as a powerful gene amplification tool due to its simplicity, speed, specificity and cost-effectiveness. This technique is being used increasingly for rapid detection and typing of emerging viruses [5], [6], [7], [8], [9], [10]. The detection of EV71 by RT-LAMP with RNA extraction was developed recently in our laboratory [11] and other groups [12], [13], [14].

Nucleic acid extraction is the first step in many molecular biology experiments, such as PCR, real-time PCR and LAMP, and is a process that has not been altered for many years. Therefore, a number of commercial kits have been developed to extract nucleic acid from different types of specimens. In order to reduce sample processing, time and cost, direct pathogen detections without nucleic acid extraction by real time PCR and LAMP in blood or serum [15], [16], [17], urine [15], cerebrospinal fluid [18], [19] and feces [20], tissue [21], cell culture supernatant [22], nasal swab [23] were reported. In this proof-of-concept study, by using a simple heat-treatment of the samples, a direct RT-LAMP assay was first developed and further evaluated for the rapid and specific detection of EV71 directly from 145 nasopharyngeal swab specimens without upstream RNA extraction by commercial kits. In parallel, the same nasopharyngeal swab specimens with RNA extraction were re-tested by both RT-LAMP and qRT-PCR. The detection results from direct RT-LAMP, RT-LAMP and qRT-PCR assays were compared.

## Materials and Methods

### Virus

EV71 isolate (Strain FY17.08/AN/CHN/2008, GenBank accession no. EU703812) with an infectivity titer of 10^6.5^ 50% tissue culture infective doses (TCID_50_)/ml on human rhabdomyosarcoma (RD) cells was served as a reference virus. Field isolates of human enterovirus known to be genetically related to HFMD were used as control viruses to evaluate the specificity of direct RT-LAMP assay for EV71. The control viruses included Coxsackievirus A (CVA) viruses (CVA 2,4,5,7,9,10,14, 16, and 24), Coxsackievirus B(CVB) viruses (CVB 1,2,3,4, and 5) and ECHO viruses (ECHO 3,6,11, and 19). One EV71-negative stool sample collected from other HFMD patients was used as a negative control. All isolates were obtained from National Laboratory for Poliomyelitis, National Institute for Viral Disease Control and Prevention, Chinese CDC, and had been verified previously by qRT-PCR and sequencing.

### Clinical samples

A total of 145 nasopharyngeal swab samples from suspicious patients with HFMD between 1 month and 9 years old enrolled in Shijiazhuang, Hebei, China in 2011 were collected.

All aspects of the study were performed in accordance with the national ethics regulations and approved by the Institutional Review Boards of the Centre for Disease Control and Prevention of China, as well as the Ethics Committee of the Centre for Disease Control and Prevention of Hebei Province. Participants were received “Written Informed Consent” on the study's purpose and of their right to keep information confidential. Written consent was obtained from their parents or grandparents.

### Design of EV71-specific RT-LAMP primers

As described previously [11], the VP1 gene of enterovirus was used to distinguish enterovirus serotypes and the primers were designed using the online software program PrimerExplorer V4 (http://primerexplorer.jp/e/).

### RNA extraction

Total RNA was extracted from 140 µl of the various nasopharyngeal swab samples or culture supernatants using QIAampViral RNA Mini Kit (Qiagen, Hilden, Germany) according to the manufacturer's instructions. The RNA was eluted in a final volumn of 50 µl RNase-free water and stored at −80°C until use.

### RT-LAMP and qRT-PCR with extracted RNA

RT-LAMP was performed as described previously [11] except for using LoopAmp RNA amplification kit and fluorescent detection reagent (FDR) (Eiken Chemical Co., Ltd., Tokyo, Japan) in accordance with the manufacturer's instructions. The RT-LAMP reaction was incubated in a Loopamp turbidimeter LA-320C (Teramecs, Japan) for real-time monitoring of the amplification at 65°C for 45 min. Positive reactions were defined as those samples having a threshold value of greater than 0.2 or a color change from faint orange to yellowish green. QRT-PCR assay was performed in an ABI Real-Time System 7500 device (Applied Biosystems, USA) with same amount of viral RNA used in RT-LAMP assay using commercial qRT-PCR Diagnostic Kits (PCR-Fluorescence Probing) for EV71 (Kinghawk Phamaceutical, China) approved by the State Food and Drug Administration of China according to the manufacturer's instructions. The qRT-PCR results were defined as the positive for Ct not higher than 37. Positive and negative controls were included in each run, and all precautions to prevent cross-contamination were conducted.

### Direct RT-LAMP with heat-treated samples without RNA extraction

For sample treatment, 8 µl of each raw sample was mixed with 12.5 µl of the LoopAmp RNA amplification reaction mix (Eiken Chemical Co., Ltd., Tokyo, Japan), 0.5 μl RNase-free water, and 0.5 μl of each primer (F3 and B3: 20 pmol/µl; BIP and FIP: 120 pmol/µl; Loop-1 and Loop-2: 60 pmol/µl). The mixture was heated at 95°C for 30 sec in a water bath and placed on ice for 2 min, 1 μl of the enzyme mix (Eiken Chemical Co., Ltd., Tokyo, Japan) was then added to the reaction mixture. Direct RT-LAMP was performed the same as RT-LAMP described above except the incubation time was extended to 75 min and 1 μl of 1∶100 diluted SYBR green I (Invitrogen, Eugene Oregon, USA) was added after amplification for the observation under the UV light by naked eyes. Positive reactions were defined as those samples having a threshold value of greater than 0.2 or a color change from orange to green fluorescence.

### Specificity and sensitivity of the RT-LAMP and the direct RT-LAMP

The specificity and sensitivity of RT-LAMP using in-house reaction buffer were described previously [11]. Similarly, the specificity and sensitivity of both the RT-LAMP and the direct RT-LAMP were performed using the Loopamp RNA amplification kit and the fluorescent detection reagent (FDR) (Eiken Chemical Co., Ltd., Tokyo, Japan) in accordance with the manufacturer's instructions with the exception of the addition of a 1∶100 diluted SYBR Green I (Invitrogen, Eugene Oregon, USA) in place of the FDR after the amplification for the direct RT-LAMP assay. RT-LAMP was tested by the use of 2 µl of extracted RNA from an EV71 reference virus or various control viruses for specificity analysis and 10-fold dilutions of a titrated EV71 for sensitivity analysis while direct RT-LAMP was tested by the use of 8 µl of heat-treated culture supernatants from the same viruses.

## Results

### Optimization of the direct RT-LAMP assay

**Figure 1:**
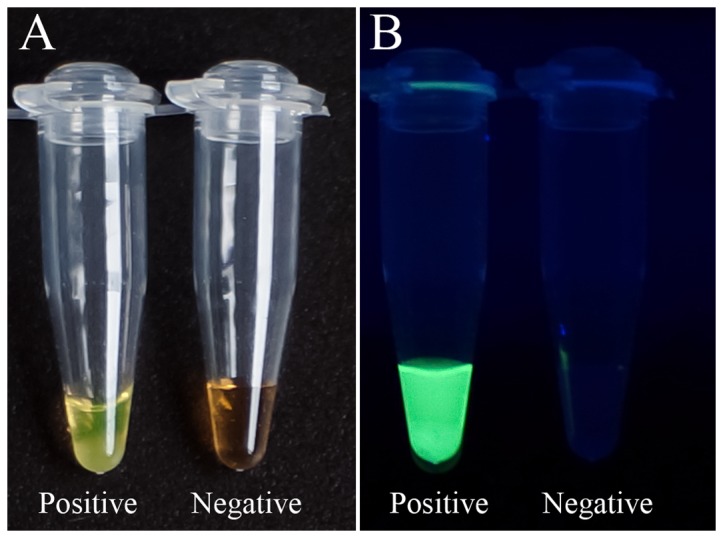
Visual detection of amplified LAMP products using SYBR green I. Addition of 1 µl of diluted SYBR green I to the reaction tube after LAMP reaction enables visible analysis of the results under natural light (Figure 1A) or UV irradiation (Figure 1B). The color changes from orange (negative reaction) to green (positive reaction) (Figure 1A) and bright fluorescence indicates a positive reaction (Figure 1B).

The direct RT-LAMP assay was performed using cell cultured reference or control viruses as templates to determine the optimal heat-treatment temperature and time, primer working concentrations and duration of the assay. The LAMP product was detected after 75 min at 65°C. After addition of 1 µl of diluted SYBR green I to the reaction tube, positive reactions (amplified products) turned green, whereas all negative controls remained orange, the starting color of SYBR green I (Figure 1).

### Specificity and sensitivity analyses of RT-LAMP and direct RT-LAMP

**Figure 2:**
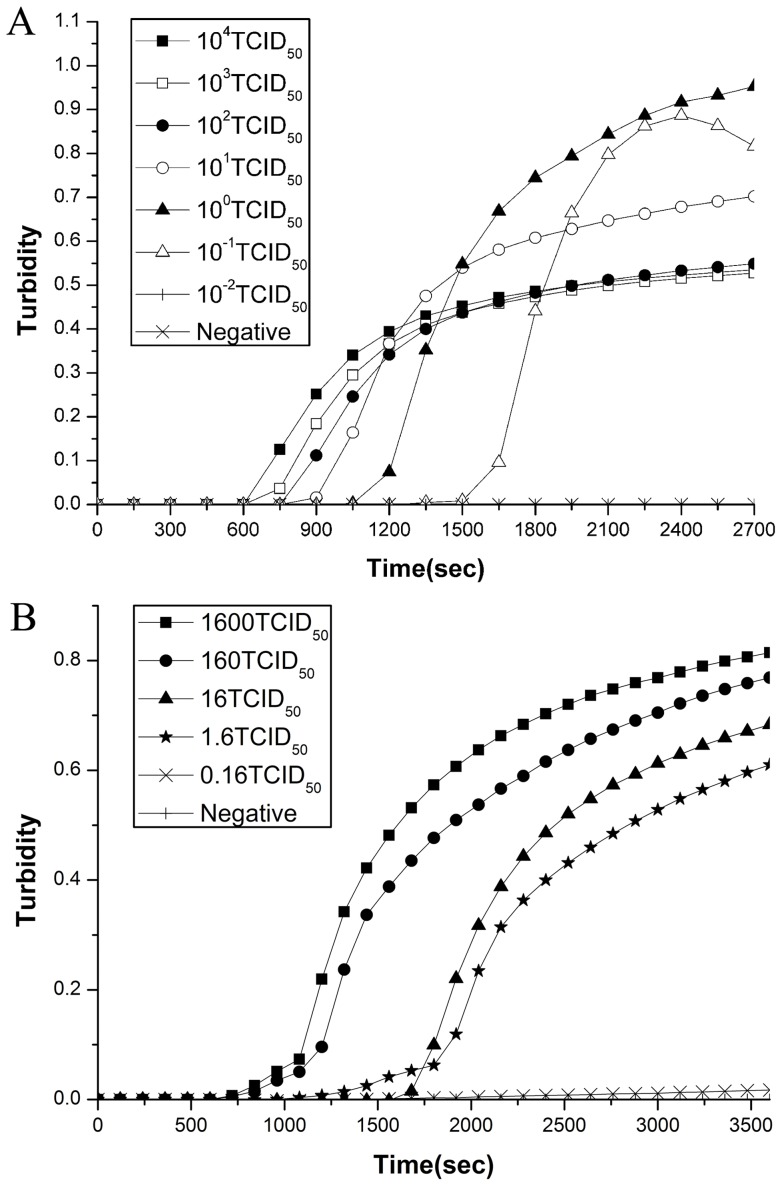
Sensitivities of the RT-LAMP and direct RT-LAMP assays for the detection of human EV71. The assay was carried out using different concentrations of RNA from a titrated EV 71 subgenotype C4 isolate (Strain FY17.08/AN/CHN/2008, GenBank accession no. EU703812) and monitored by real-time measurement of turbidity. The detection limits of the RT-LAMP (Figure 2A) and direct RT-LAMP (Figure 2B) assay for EV71 were 0.1 and 1.6 TCID_50_ per reaction, respectively.

The sensitivity and specificity of both RT-LAMP and direct RT-LAMP were compared. The detection limits of the RT-LAMP and direct RT-LAMP assays were 0.1 and 1.6 of a 50% tissue culture infective dose (TCID_50_) per reaction based on 10-fold dilutions of a titrated EV71 reference virus (Figure 2). No cross-reaction was observed with control viruses including Cosackievirus A (CVA) viruses (CVA2,4,5,7,9,10,14,16, and 24), Coxsackievirus B(CVB) viruses (CVB1,2,3,4, and 5) or ECHO viruses (ECHO3,6,11, and 19) for both RT-LAMP and direct RT-LAMP assays (data not shown).

### Evaluation of direct RT-LAMP assay with clinical specimens and comparison with RT-LAMP and qRT-PCR assays

A total of 145 heat-treated nasopharyngeal swab samples from suspicious HFMD patients were tested by direct RT-LAMP. In parallel, same amount of extracted RNA were tested by both RT-LAMP and qRT-PCR. As summarized in Table 1, the direct RT-LAMP assay and the RT-LAMP assay were in complete agreement for 133/145 (91.7%) of the specimens, while in Table 2, the direct RT-LAMP assay and the qRT-PCR assay were in complete agreement for 128/145 (88.3%) specimens. No false positive was found with either RT-LAMP or direct RT-LAMP. The sensitivity and specificity of the direct RT-LAMP was 90.3% and 100% respectively, compared to RT-LAMP, and 86.8% and 100% respectively, compared to qRT-PCR.

**Table 1:** Comparison of results of direct RT-LAMP assay with RT-LAMP assays obtained from 145 clinical nasopharyngeal swab specimens.

Direct RT-LAMP	RT-LAMP^a^	
	Positive	Negative
Positive	112	0
Negative	12	21

a A total of 145 clinical nasopharyngeal swab specimens detected by RT-LAMP included 124 EV71 positive and 21 EV71 negative samples.

**Table 2:** Comparison of results of direct RT-LAMP assay with qRT-PCR assays obtained from 145 clinical nasopharyngeal swab specimens.

Direct RT-LAMP	qRT-PCRa,b	
	Positive	Negative
Positive	112	0
Negative	17	16

a A total of 145 clinical nasopharyngeal swab specimens detected by qRT-PCR included 129 EV71 positive and 16 EV71 negative samples.

b Of 17 positive samples detected by qRT-PCR, 12 samples were positive by RT-LAMP.

## Discussion

Rapidity and simplicity of the methods for the EV71 detection are critical for community hospital laboratory or field use, RT-LAMP assays with RNA extraction for the rapid diagnosis of EV71 infection have been reported previously [11], [12], [13], [14]. As the RNA extraction step requires approximately 30 min, omission of RNA extraction could save both time and labor for preparing the samples for RT-LAMP, a major advantage for rapid diagnosis in hospital laboratories or field use.

To further reduce the cost and turnaround time of the RT-LAMP assay, the feasibility of direct RT-LAMP assay without upstream RNA extraction was investigated in this study. Briefly, 8 µl of raw sample with reaction mixture was heated at 95°C for 30 sec, and detected directly by the RT-LAMP assay. The result of direct RT-LAMP assay was monitored by a real-time turbidity meter or by direct visual inspection under UV light after amplification.

The proposed method of sample heat-treatment was highly optimized in terms of the reaction buffer. Several other pretreatment methods without RNA extraction using various lysis buffers other than the reaction mixture as described in this study were also examined. In our preliminary study, 50 µl of each raw sample (5 EV71-positive and 1 EV71-negative sample) was mixed with either 100 µl of lysis buffer A containing 5% NP-40 and 1.5% 2-mercaptoethanol, or lysis buffer B containing 0.1 M Tris-HCl and 0.05% Tween 20, or lysis buffer C containing 0.1 M Tris-HCl, 0.05% Tween 20 and 0.24 mg/ml proteinase K. After incubation at room temperature for 15 min, the mixture was heated at 75°C for 5 min in a water bath and 8 µl of each supernatant was added to the reaction mixture for RT-LAMP. However, none of these attempts using lysis buffer A or B or C produced better detection results in comparison with the result of heat-treatment using reaction mixture directly (Table 3). The heat-treatment temperature and incubation time were also optimized compared with reported direct LAMP assays for DNA viruses such as the herpes simplex virus [24], human herpesvirus 6 [25], [26] and equine herpesvirus 1 [23] since the great difference of thermal stability between DNA and RNA [27].

**Table 3:** Preliminary study of sample treatment for EV71 detection by direct RT-LAMP using various lysis buffers and heat-treatment.

Sample^a^	Time^b^(min)			
	Lysis buffer A^c^	Lysis buffer B^c^	Lysis buffer C^c^	Heat- treatment^c^
BF-07	25	46	46	25
BF-09	36	55	53	28
WG12	47	53	52	30
XH14	45	46	48	29
XH106	36	47	51	22
JX49	ND^d^	ND	ND	ND

a A total of 5 EV71-positive swab samples (BF-07, BF-09,WG12, XH14, XH106) and 1 EV71 negative swab sample (JX49) were included in this preliminary study.

b The time (minute) for those samples reaching a threshold value of greater than 0.2 or having a color change from orange to green.

c Sample pretreatment methods using different lysis buffers and heat-treatment in the text.

d ND: Not detect.

To evaluate the direct RT-LAMP assay, RNAs from clinical nasopharyngeal swab samples were extracted using commercial RNA extraction kit and re-tested by both qRT-PCR and RT-LAMP. Results from the direct LAMP and qRT-PCR or RT-LAMP assay were compared. The overall performance of qRT-PCR and RT-LAMP was comparable (96.5% accordance rate). Of 129 nasopharyngeal swab samples that were positive in qRT-PCR, 5 were negative in RT-LAMP assay and 17 were negative in direct RT-LAMP (Table 1 and 2). Among the 17 negative samples using direct RT-LAMP, 14 samples had high Ct values (>34) and the Ct values for other 3 samples ranged from 27 to 31 (29 in average). The discrepant detection result from qRT-PCR and direct RT-LAMP is due to the slightly less sensitivity of direct RT-LAMP attributable to the following factors: 1) reduced sample input (8 µl of raw sample), 2) the lower viral load in the sample, 3) varied sample quality (the potential amplification inhibitor present in some raw samples). As no false-positive was detected by direct RT-LAMP, our results of clinical utility suggested direct RT-LAMP assay is simple to perform without compromising significantly its sensitivity and specificity. In addition, of 145 nasopharyngeal swab samples, 72 heat-treated samples were also tested similarly by the direct qRT-PCR, only about 60% accordance rate was achieved compared to the qRT-PCR, suggesting Bst DNA polymerase is more tolerable to the inhibitors than Taq polymerase in the amplification of the raw samples (data not shown).

The use of the heat-treatment method for template preparation provides a good alternative to the expensive and labor intensive RNA isolation methods that might not always be possible in field settings. Direct amplification from heat-treated nasopharyngeal swab samples in combination with assessment by turbidity assay or by the use of SYBR green I for visual observation would accomplish the entire amplification within 75 min (most positive reactions occurred within 45 min). This system would therefore allow large decreases in cost and time, making it more attractive for clinical laboratory use or field use.

To our knowledge, the proposed method is the first report to detect EV71 in nasopharyngeal swab specimens using direct RT-LAMP. The RT-LAMP assay with heat-treatment is easy to perform and so should be a potentially useful tool for routine diagnosis of EV71 infection in clinical samples and improves the effectiveness of local surveillance programs to control the spread of HFMD outbreaks. Further improvement by large-scale studies for determination of the sensitivity, specificity, and clinical utility of this new method will be needed before this method can find wider clinical applicability.
